# Transmitting Status Updates on Infinite Capacity Systems with Eavesdropper: Freshness Advantage of Legitimate Receiver

**DOI:** 10.3390/e27060571

**Published:** 2025-05-27

**Authors:** Jixiang Zhang, Han Xu, Anqi Zheng, Daming Cao, Yinfei Xu, Chengyu Lin

**Affiliations:** 1 School of IoT Engineering, Wuxi Taihu University, Wuxi 214063, China; zhangjx@wxu.edu.cn (J.Z.); zhengaq@wxu.edu.cn (A.Z.); 2 Provincial Key Laboratory of Intelligent Internet of Things Technology and Applications, Wuxi 214063, China; 3 School of Information Science and Engineering, Southeast University, Nanjing 210096, China; han_xu@seu.edu.cn (H.X.); yinfeixu@seu.edu.cn (Y.X.); 4 School of Electrical and Information Engineering, Nanjing University of Information Science and Technology, Nanjing 210044, China; dmcao@nuist.edu.cn

**Keywords:** age of information, freshness advantage, infinite capacity system, transmission timeliness, transmission security, wiretap channel

## Abstract

We consider the scenario in which the source sends status updates, or packets, to the receiver through an infinite capacity transmitter, where the transmitted packets are subject to potential illegal eavesdropping. Time is discretized into identical time slots. In recent years, the age of information (AoI) metric, which was defined as the time has elapsed since the generation instant of the latest received packet, has been widely applied to characterize the freshness of obtained packets. Due to the presence of eavesdroppers, some packets may be eavesdropped during their transmissions, causing information leakages. To assess an infinite-capacity system’s performance of securely transmitting status updates, in this paper, we define an AoI-related metric called the *freshness advantage* of the legitimate receiver, *F*, to be average instantaneous gap between eavesdropper’s and legitimate receiver’s AoI. For arbitrarily distributed packet interarrival times, and assuming that in each time slot with probabilities $\gamma_{d}$, $\gamma_{E}$, the transmitted packet is received by the legitimate receiver and the eavesdropper, we derive the explicit formula of *F*. The concise expression shows that *F* is fully determined by the average interarrival time and the ratio of $\gamma_{d}$ to $\gamma_{E}$. For special cases where the interarrival time follows geometric distributions, we first determine the explicit distribution of instantaneous AoI gap. Then, given $\gamma_{d}$ and $\gamma_{E}$, we derive the optimal packet generation rate $p^{*}$ that minimizes the combined performance *Q*, which is constructed as the average AoI minus the freshness advantage *F*. When imposing timeliness and security constraints at the same time, the feasible regions of *p* and $\gamma_{d}$ such that both two required performances can be satisfied are depicted and discussed. Finally, we investigate the impacts of different parameters on *F* and show the tradeoffs between timeliness performance and security performance through numerical simulations.

## 1. Introduction

Timely transmitting information plays an important role in the Internet of Things (IoT) applications and modern communication systems. In various applications on IoT networks, real-time transmission ensures the immediacy and accuracy of data between devices, which is crucial for fields such as autonomous driving, intelligent healthcare, and smart cities. For example, traffic monitoring systems in smart cities need to upload real-time traffic data to adjust traffic signals and route planning in a timely manner, which enhances traffic efficiency and safety. In modern communication systems such as 5G/6G systems, real-time transmission is also very important to ensuring normal system operations and improving user experience. It enables participants to interact in real-time without waiting for data transmission, especially for situations where quick responses are required.

In paper [1], Kaul and Yates et al. modeled a status updating system and proposed the age of information (AoI) metric to characterize the freshness of packets when they arrive at the receiver side. Since then, for different kinds of queues, stationary average AoI and its distribution property of various status updating systems has been investigated by researchers from multiple communities. According to transmission system’s structure, the average AoI of some basic systems, for example, system with M/M/1/1, M/M/1/2, M/M/1/*∞* queues and their variants, were determined in works [2,3,4,5]. Then, age analysis was extended to more general status updating systems who has multiple source nodes and/or transmitters. To model multiple access situations in network communications, in papers [6,7,8], the authors considered a transmission scenario where multiple sources communicate with the receiver through one common transmitter. Assuming that the system applies different service disciplines, such as First-Come First-Serve (FCFS), Last-Come First-Serve (LCFS) with/without packet preemption, average AoI of each source was obtained. On the other hand, considering different control schemes such as priority strategy and packet deadline policy, each source’s average AoI in multi-source status updating systems was studied in works [9,10,11,12,13,14]. For systems with several parallel servers which model packet transmissions via multiple paths, characterizing AoI performance is more complex. Multi-path transmission makes it difficult to analyze AoI evolutions, as packets may not arrive at the receiver in chronological order that they were generated at the source. AoI of multi-server system was investigated in works [15,16,17]. AoI analysis reaches its peak when discussing average version age in a node of gossip networks. Timely transmitting status updates on gossip network was first studied by Yates in [18,19] and was extended by Buyukates and Ulukus et al. in subsequent works [20,21,22,23]. Apart from the above works, as an information freshness metric, AoI was considered in many resource scheduling and communication system design problems as well, for example, in papers [24,25,26,27,28,29,30]. Different from analyzing AoI in continuous time systems, the mean and the distribution properties of discrete-time AoI have also received significant attention in recent years. Studies considering discrete time AoI include [31,32,33,34], as well as our works in [35,36,37,38].

Recently, the issue of securely transmitting packets in real-time communication scenarios has attracted the attention of many researchers. Yang [39,40] considered a classical three-node wiretap channel. Assuming that there exists an eavesdropper, the author discussed transmission security perfomance of the system when the packets are transmitted based on a short-packet permutation-based protocol. Yang defined *average secrecy margin* to be a transmission security metric, which denotes the average area of a random region enclosed by AoI’s sample path and the time axis, from the instant that the legitimate receiver obtains a packet to the instant that the eavesdropper obtains it. A larger average secrecy margin means the transmission security of the system is higher, because the larger the region represented by average secrecy margin, the more likely a legitimate receiver will obtain the transmitted packet earlier than the eavesdropper. Within the paper, the author derived the optimal packet length such that the average secrecy margin is maximized. In reference [41], Wang and Chen et al. considered the same communication scenario. Unlike Yang’s work, they defined the *secrecy age* as instantaneous difference between eavesdropper’s AoI and legitimate receiver’s AoI, and *secrecy age outage probability* as the probability that the real time gap between two AoIs is less than some given threshold. On discrete-time communication models, for stationary random and threshold based packet generation strategies, the authors determined the accurate expressions of average secrecy age and average secrecy age outage probability of three-node wiretap transmission system. By jointly optimizing two security metrics, they derived the optimal packet generation rate that maximizes the security performance. Within the framework of AoI, simultaneously characterizing and designing transmission timeliness and transmission security has become a new research hotspot. In paper [42], the authors analyzed the average age of secure packets. They ignored all of insecure packets when those packets arrive at the receiver. The paper [43] considered the situation in which the eavesdropper is an energy harvesting node, and the authors discussed the optimal transmission strategy for minimizing average age of secure information (AoSI). Apart from these, from the perspective of secure transmission at physical layer, using Wyner secrecy capacity [44] as transmission security metric, simultaneously designing timeliness and security of some communication systems have been discussed in many works, for example, in [45,46,47,48] and some cited references of those works. In addition, securely transmitting data against the eavesdroppers was also investigated in intelligent transmission systems, such as mmWave vehicular networks, in papers [49,50].

In this paper, we characterize the performance of infinite capacity systems securely transmitting packets within the framework of AoI. We propose *freshness advantage F* of legitimate receiver as the metric to assess system’s transmission security. Similar to [41], freshness advantage *F* is constructed to be average instantaneous gap between eavesdropper’s and legitimate receiver’s AoI. However, we point out that the security metric proposed in paper [41] requires that the instantaneous AoI of eavesdropper must be greater than that of legitimate receiver, meaning that the constructed security metric takes only positive values (so does Yang’s work [39,40]). In contrast, we allow the instantaneous AoI of eavesdropper to be smaller than that of legitimate receiver, which implies that in some cases our security metric *F* can take negative values. Through the general formula derived in this paper, we will demonstrate that taking negative security performance into consideration will yield more comprehensive results. Another method of constructing transmission security metric is calculating the ratio of eavesdropper’s and legitimate’s AoI. Requiring that the eavesdropper’s AoI is less than certain threshold, from the perspective of game theory maximization of this ratio was investigated in paper [51]. By a Bergson social welfare framework, the author solved the problem of finding the optimal generation rate as a function of probability of data capture by the eavesdropper.

Another major difference between this work and the existing literatures lies at that we consider infinite capacity status updating systems, while those works discussed bufferless or finite capacity systems. The reason for considering infinite capacity transmission systems are twofold. Firstly, in some practical scenarios, assuming that the system has infinite capacity provides a simple modeling method. For example, when discussing timely source coding problem in [52,53], Yates et al assumed that the transmitter has an infinite capacity buffer such that all of packets that have not been transmitted can be stored in the system. On the other hand, the performance of infinite capacity systems can serve as a reference for the performance of finite capacity systems. For example, in paper [14] the average AoI of any finite capacity system with packet deadline will not be greater than that of an infinite capacity system with the same packet deadline. Therefore, the known average AoI of infinite capacity system provides an upper bound on the average AoI of any finite capacity systems.

Within the framework of AoI, assuming that there exists a passive eavesdropper, this paper considers the performance of infinite capacity status updating system securely transmitting packets. We define freshness advantage *F* to evaluate the transmission security, which is equal to average instantaneous gap between eavesdropper’s AoI and legitimate receiver’s AoI. The main contributions of the paper include the following aspects:

1. For arbitrary packet arrival processes, we derive the explicit expression of freshness advantage *F* and prove that *F* is completely determined by average packet interarrival time and the ratio of $\gamma_{d}$ and $\gamma_{E}$;
2. Assuming that packet arrivals form the Bernoulli(p) process, we determine the specific distribution of instantaneous gap between two AoIs;
3. By constructing the combined performance *Q* to be average AoI minus *F*, we derive the optimal packet generation rate $p^{*}$ such that *Q* is minimized, such that investigating the tradeoffs between the timeliness and security;
4. In case of Bernoulli packet arrivals, and assuming that timeliness and security are both limited, we depict and analyze the feasible region composed of *p* and $\gamma_{d}$.

The rest of the paper is organized as follows. In Section 2, we introduce the model of considered transmission system and define the proposed security metric, that is, the freshness advantage of legitimate receiver *F*. In Section 3, we first derive the explicit formula of *F*, then calculate the specific distribution of age gap *g* when packet arrivals form the Bernoulli(p) process. Joint optimization of timeliness and security are investigated in Section 4, where we define the combined performance metric *Q* as average AoI minus *F* and derive the optimal *p* such that *Q* is minimized. In addition, under the condition that both average AoI and *F* are limited, we also depict and discuss the feasible region composed of *p* and $\gamma_{d}$ in Section 4. Numerical simulations are provided in Section 5 to demonstrate the results obtained in the paper. In particular, we draw the average AoI-*F* curves to demonstrate the direct tradeoffs between two performances. We finally conclude the paper and discuss some possible further work in Section 6.

## 2. System Model and Problem Formulation

**Figure 1:**
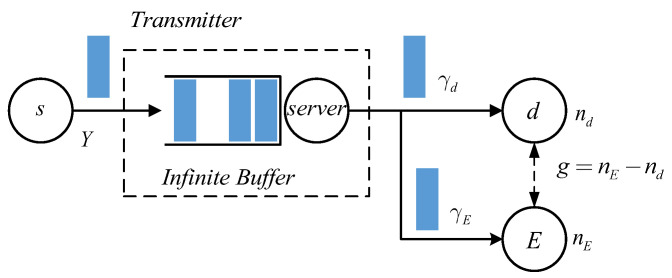
Model of infinite capacity status updating system with an eavesdropper: characterizing instantaneous gap *g* between eavesdropper’s AoI $n_{E}$ and legitimate receiver’s AoI $n_{d}$.

We depict the model of an infinite capacity status updating system in Figure 1. The transmission model and considered problem are explained as follows.

Considering the scenario where the source node *s* sends packets to the receiver *d* through an infinite capacity transmitter. Denote *Y* to be i.i.d. interarrival time between two successively generated packets. Following the First Come-First Serve (FCFS) discipline, these packets are delivered to the receiver *d*. Assuming that the transmitter has infinite capacity so that all of newly arrived packets that are not served in time can be stored in the buffer. At receiver side, age of information (AoI) process describes the evolution of difference between current time and the generation instant of the latest obtained packet. The value of AoI increases 1 after each time slot if no newer packet is obtained at the receiver. On the contrary, every time when a fresher packet, who has smaller age, arrives to *d*, the value of AoI decreases to instantaneous age of that packet, and we call these age reduction operations as AoI updatings. Let Δ(k) be the value of AoI process at the *k*th time slot, we can describe above AoI evolutions mathematically by

(1) $$\Delta \left(k+1\right)=\left\{\begin{array}{cc} \Delta (k)+1, & d\;\mathrm{obtains}\;\mathrm{no}\;\mathrm{packet}\\ \Delta (k)+1-Y, & d\;\mathrm{obtains}\;a\;\mathrm{newer}\;\mathrm{packet} \end{array}\right.$$

where *Y* is interarrival time between the last obtained packet and the current arrived one. Random variable *Y* characterizes the behavior of packet generations or packet arrivals. For the case where packet generations form a Bernoulli process, inter-arrival time *Y* follows an i.i.d. geometric distribution. Since the transmitter has infinite capacity, notation *Y* in Equation (1) is the same as that *Y* we depict in Figure 1. Stationary average AoI and other AoI-related quantities, such as average peak AoI and AoI-violation probability, have been widely used to characterize status updating system’s timeliness of transmitting packets. Apparently, a lower average AoI implies that the system has better transmission timeliness.

During packet transmissions, it is assumed that a passive eavesdropper *E* attempts to obtain the packets and steal the contained information illegally. Due to signal fadings and the unreliability of wireless channels, in the considered model we assume that in each time slot, a transmitted packet is obtained at legitimate receiver with probability $\gamma_{d}$ and the successful eavesdoppering probability at *E* is equal to $\gamma_{E}$. In general cases, we have $\gamma_{d}>\gamma_{E}$, that is, the probability of transmitting a packet to legitimate receiver is higher than that of being eavesdropped.

In status updating systems, notice that as long as a packet is successfully sent to the receiver, the source will not transmit this packet again. Therefore, in order to improve the security of transmitting status updates, we should try to make legitimate receiver obtain the packet earlier than eavesdropper (Strictly speaking, requiring legitimate receiver obtaining packets before eavesdropper cannot guarantee that the transmitted status updates are absolutely secure. Imagining that in general network communication scenarios, the same message may be transmitted to the receiver through multiple paths. When eavesdropper gets a packet later than legitimate receiver from one path, and the same packet is the newest one that legitimate receiver currently owns, which has not been updated by fresher packets from the source. In this case, legitimate receiver’s packet becomes insecure. The current paper only discusses point-to-point transmission scenarios. Since a packet is never retransmitted once it is obtained by legitimate receiver, thus as long as a packet is transmitted to legitimate receiver earlier than eavesdropper, in point-to-point communication scenarios, this packet is absolutely secure. Those more general network communication situations and AoI-based transmission security will be interesting topics for future research). Applying AoI metric, we define freshness advantage *F* of legitimate receiver as the long-term average gap between instantaneous AoI of eavesdropper and that of legitimate receiver, i.e.,

(2) $$\begin{array}{cc} F & =\lim_{K\to \infty }\frac{1}{K}\sum_{k=1}^{K}\left[n_{E}\left(k\right)-n_{d}\left(k\right)\right] \end{array}$$

(3) $$\begin{array}{cc} \; & =E[n_{E}-n_{d}] \end{array}$$

which is used to measure the transmission security of status updating systems. Notice that in Figure 1, we have represented this instantaneous gap with *g*, that is, we denote $g=n_{E}-n_{d}$. In obtaining (3), we have assumed that the evolution process of two AoIs’ gap is ergodic.

In following sections, we will derive the explicit formula of *F* for infinite capacity status updating system, investigate how different system parameters affect *F*, and discuss the trade-offs between transmission timeliness and transmission security, which are represented by average AoI and *F*.

## 3. Freshness Advantage of Legitimate Receiver of Infinite Capacity Status Updating Systems

By constructing a two-dimensional random process to simultaneously describe the evolutions of AoI and the age of served packet, we have derived the stationary distribution and the mean of infinite capacity system’s AoI in previous work [36]. Taking eavesdropping into consideration, of course it is feasible to characterize the evolutions of instantaneous gap $g=n_{E}-n_{d}$, if we define a third-dimensional process, say $\left\{\left(n_{E,k},n_{d,k},m_{k}\right):k\in N\right\}$, to track random transfers of two AoIs and age of transmitted packet *m* at the same time. However, it turns out that using three-dimensional stochastic process involves a significant amount of computation, making the problem more complex. In the following, we will show that characterizing the random gap itself is actually simpler, and it is sufficient to use a one-dimensional process.

### 3.1. Freshness Advantage in General Packet Arrival Process

In describing random transfers of age gap *g*, we use the assumption that the server is never empty. That is, when current packet completes its transmission, there always exists some new packets in the buffer such that the server can continue working. We point out that this assumption is reasonable, because it is unnecessary to consider the time period without packet transmission when characterizing transmission security of a system.

Define two binary random variables $B_{d}$, $B_{E}$ to indicate in a time slot, if the transmitted packet is obtained by the receiver and the eavesdropper. Dividing into three cases, i.e., g>0, g=0, and g<0, we summarize the random transfers of age gap *g* in Table 1 and explain them as follows.

Notice that as long as the packets owned by legitimate receiver and the eavesdropper do not change, i.e., neither of them are updated by fresher packets from the source, the difference between two AoIs remains unchanged. This is because as time slot passes, both of two AoIs increase synchronously. Therefore, age gap evolution depends entirely on the situations that the legitimate receiver and the eavesdropper obtain the packets. According to different realizations of $B_{d},B_{E}$, it divides into four cases. Firstly, we consider two simple situations. If $B_{d}=B_{E}=0$, which means neither of receiver and the eavesdropper obtain the packet, then *g* is unchanged. While if $B_{d}=B_{E}=1$, indicating that both of two nodes receive the transmitted packet, in this case *g* changes to zero become the receiver and the eavesdropper obtain the same packet. Next, assume that $B_{d}=1$ and $B_{E}=0$. It represents that the legitimate receiver obtains the packet but the eavesdropper does not. At this time, within the age gap $g=n_{E}-n_{d}$, $n_{d}$ decreases to the age of newly received packet, thus *g* increases and the random increment is exactly equal to interarrival time *Y*. For last case of $B_{d}=0,B_{E}=1$, that is the eavesdropper obtains the packet while the legitimate receiver has not. In this case, for age gap $g=n_{E}-n_{d}$, $n_{E}$ is reduced to new packet’s age. It sees that *g* decreases to some *negative* values depending on different realizations of *Y*, because the eavesdropper obtains a newer packet that has not been transmitted to legitimate receiver, causing $n_{E}<n_{d}$. Starting from a negative *g* (notice that a negative *g* only occurs when eavesdropper obtains the transmitted packet earlier than legitimate receiver, thus when determining random transfers from negative *g*’s, we only need to consider $B_{d}$), for the case of $B_{d}=0$, *g* does not change, since the packets owned at both nodes are not updated. Until the legitimate receiver obtains the packet that has been received by the eavesdropper, *g* returns to zero.

**Remark** **1.** *After describing state transfers of age gap g, now we explain why the assumption that the server is never empty is necessary. In the cases where a packet is successfully transmitted to the receiver and there are no packets in transmitter’s buffer, the server will become idle. During the period when the server is waiting for next new packet, age gap $g=n_{E}-n_{d}$ remains unchanged because the packets owned by legitimate receiver and eavesdropper will not be updated, and the instantaneous age of these packets increases synchronously. Within these idle periods, the state transfer described in Table 1 is actually paused and restarts until the next new packet enters the server. It shows that the existence of server idle periods makes the random state transfers in Table 1 not continuous, but intermittent. When calculating time average g, g will also be averaged over these idle periods. However, characterizing the accurate distribution properties of these idle periods is not easy. Therefore, assuming that the server is never empty simplifies the calculation of average age gap, i.e., freshness advantage F. At the same time, from the perspective of practical applications, we point out that it is unnecessary to consider the time period during which no packets are transmitted in characterizing the transmission security of a communication system.*

After long time evolutions, assuming that age gap process reaches steady state. For each g∈Z, define $\pi_{g}$ as corresponding stationary probability. From random transfers and their transition probabilities given in Table 1, we establish follwoing stationary equations.

(4) $$\left\{\begin{array}{cc} \pi_{g}=\pi_{g}\left(1-\gamma_{d}\right)\left(1-\gamma_{E}\right)+\pi_{0}\gamma_{d}\left(1-\gamma_{E}\right)\mathrm{Pr}\left\{Y=g\right\}\\ \;\;+\sum_{k=1}^{g-1}\pi_{k}\gamma_{d}\left(1-\gamma_{E}\right)\mathrm{Pr}\left\{Y=g-k\right\} & \;(g\geq 2)\\ \pi_{1}=\pi_{1}\left(1-\gamma_{d}\right)\left(1-\gamma_{E}\right)+\pi_{0}\gamma_{d}\left(1-\gamma_{E}\right)\mathrm{Pr}\left\{Y=1\right\}\\ \pi_{0}=\pi_{0}\left[\left(1-\gamma_{d}\right)\left(1-\gamma_{E}\right)+\gamma_{d}\gamma_{E}\right]\\ \;\;+\sum_{j=-\infty }^{-1}\pi_{j}\gamma_{d}+\sum_{k=1}^{+\infty }\pi_{k}\gamma_{d}\gamma_{E}\\ \pi_{g}=\pi_{g}\left(1-\gamma_{d}\right)+\pi_{0}\left(1-\gamma_{d}\right)\gamma_{E}\mathrm{Pr}\left\{Y=|g|\right\}\\ \;\;+\sum_{k=1}^{+\infty }\pi_{k}\left(1-\gamma_{d}\right)\gamma_{E}\mathrm{Pr}\left\{Y=|g|\right\} & \;(g\leq -1) \end{array}\right.$$

Each equation in (4) shows that under the corresponding conditions, the state on the left side can be transferred to from those states on the right side.

In following paragraphs, we derive the general formula of freshness advantage *F* using stationary Equations (4). According to its definition, we have that

(5) $$\begin{array}{cc} F & =\sum_{g=-\infty }^{+\infty }g\pi_{g}\\ \; & =\sum_{g=-\infty }^{-1}g\pi_{g}+\sum_{g=1}^{+\infty }g\pi_{g}\\ \; & =\beta_{+}^{\prime }\left(1\right)-\beta_{-}^{\prime }\left(1\right) \end{array}$$

In Equation (5), for 0<z≤1 we define

(6) $$\beta_{+}\left(z\right)=\sum_{g=1}^{+\infty }z^{g}\pi_{g},\;\beta_{-}\left(z\right)=\sum_{g=-\infty }^{-1}z^{\left|g\right|}\pi_{g}$$

and $\beta_{+}^{\prime }\left(1\right)$, $\beta_{-}^{\prime }\left(1\right)$ are their derivatives at point z=1. Both functions are determined in following Lemma 1.

**Lemma** **1.** *Functions $\beta_{+}\left(z\right)$ and $\beta_{-}\left(z\right)$ are derived as*

(7) $$\begin{array}{cc} \beta_{+}\left(z\right) & =\frac{\pi_{0}\gamma_{d}\left(1-\gamma_{E}\right)Y\left(z\right)}{1-\left(1-\gamma_{d}\right)\left(1-\gamma_{E}\right)-\gamma_{d}\left(1-\gamma_{E}\right)Y\left(z\right)} \end{array}$$

(8) $$\begin{array}{cc} \beta_{-}\left(z\right) & =\frac{\left(1-\gamma_{d}\right)\gamma_{E}Y\left(z\right)}{1-\left(1-\gamma_{d}\right)\left(1-\gamma_{E}\right)} \end{array}$$

*in which $Y\left(z\right)=\sum_{j=1}^{\infty }z^{j}\mathrm{Pr}\left\{Y=j\right\}$ is probability generation function (PGF) of Y, and $\pi_{0}$ is determined to be*

(9) $$\pi_{0}=\frac{\gamma_{d}\gamma_{E}}{{\left[1-\left(1-\gamma_{d}\right)\left(1-\gamma_{E}\right)\right]}^{2}}$$

*Notice that $\pi_{0}$ is independent of Y, i.e., packet arrival process.*

**Proof.**  Lemma 1 is proved in Appendix A. □

Using the results given in Lemma 1, we calculate $\beta_{+}^{\prime }\left(1\right)$ and $\beta_{-}^{\prime }\left(1\right)$ as follows. Firstly, Equation (7) shows that

(10) $$\begin{array}{c} \beta_{+}\left(z\right)\left(1-\left(1-\gamma_{d}\right)\left(1-\gamma_{E}\right)-\gamma_{d}\left(1-\gamma_{E}\right)Y\left(z\right)\right)=\pi_{0}\gamma_{d}\left(1-\gamma_{E}\right)Y\left(z\right) \end{array}$$

Taking derivative of *z* in both sides, then letting z=1 yields

(11) $$\begin{array}{cc} \; & \beta_{+}^{\prime }\left(1\right)\gamma_{E}-\beta_{+}\left(1\right)\gamma_{d}\left(1-\gamma_{E}\right)E\left[Y\right]=\pi_{0}\gamma_{d}\left(1-\gamma_{E}\right)E\left[Y\right] \end{array}$$

where $Y^{\prime }\left(1\right)=E\left[Y\right]$ is obtained from its definition. From Equation (11) we solve that

(12) $$\begin{array}{cc} \beta_{+}^{\prime }\left(1\right) & =\frac{\left(\pi_{0}+\beta_{+}\left(1\right)\right)\gamma_{d}\left(1-\gamma_{E}\right)E\left[Y\right]}{\gamma_{E}} \end{array}$$

(13) $$\begin{array}{cc} \; & =\frac{\gamma_{d}^{2}\left(1-\gamma_{E}\right)E\left[Y\right]}{\gamma_{E}\left[1-\left(1-\gamma_{d}\right)\left(1-\gamma_{E}\right)\right]} \end{array}$$

in which

(14) $$\pi_{0}+\beta_{+}\left(1\right)=\frac{\gamma_{d}}{1-\left(1-\gamma_{d}\right)\left(1-\gamma_{E}\right)}$$

is obtained in Appendix A in Equation (A12).

Similarly, it is easy to obtain $\beta_{-}^{\prime }\left(1\right)$ from (8) as

(15) $$\beta_{-}^{\prime }\left(1\right)=\frac{\left(1-\gamma_{d}\right)\gamma_{E}E\left[Y\right]}{1-\left(1-\gamma_{d}\right)\left(1-\gamma_{E}\right)}$$

Applying above results, we derive the explicit expression of *F* in Theorem 1.

**Theorem** **1.** *Assuming that there exists passive eavesdroppers. When transmitting packets through an infinite capacity status updating system, the freshness advantage F of legitimate receiver is determined as*

(16) $$F=E\left[Y\right]\left(\frac{\gamma_{d}}{\gamma_{E}}-1\right)$$

*where Y is packet interarrival time, $\gamma_{d},\gamma_{E}$ are success probabilities of transmitting one packet to legitimate receiver and eavesdropper in a time slot.*

**Proof.**  Substituting (13) and (15) into (5), we have

$$\begin{array}{cc} F & =\beta_{+}^{\prime }\left(1\right)-\beta_{-}^{\prime }\left(1\right) \end{array}$$

(17) $$\begin{array}{cc} \; & =E\left[Y\right]\frac{\gamma_{d}^{2}\left(1-\gamma_{E}\right)-\left(1-\gamma_{d}\right)\gamma_{E}^{2}}{\gamma_{E}\left[1-\left(1-\gamma_{d}\right)\left(1-\gamma_{E}\right)\right]} \end{array}$$

(18) $$\begin{array}{cc} \; & =E\left[Y\right]\frac{\left(\gamma_{d}+\gamma_{E}-\gamma_{d}\gamma_{E}\right)\left(\gamma_{d}-\gamma_{E}\right)}{\gamma_{E}\left[1-\left(1-\gamma_{d}\right)\left(1-\gamma_{E}\right)\right]} \end{array}$$

(19) $$\begin{array}{cc} \; & =E\left[Y\right]\left(\frac{\gamma_{d}}{\gamma_{E}}-1\right) \end{array}$$

This completes the proof of Theorem 1. □

For Theorem 1, we provide following three aspects discussion.

**Remark** **2.** *Firstly, the surprisingly concise expression (16) indicates that when transmitting packets through an infinite capacity system, the freshness advantage of legitimate receiver over eavesdropper is fully determined by average packet interarrival time and the ratio $\gamma_{d}/\gamma_{E}$, and $\gamma_{d}/\gamma_{E}>1$ serves as the sufficient and necessary condition such that F>0. At the same time, it shows that F can be unbounded as E[Y] or $\gamma_{d}/\gamma_{E}$ tends to be infinite. Of course, this is unrealistic. When designing practical transmission systems, a very large E[Y] will deteriorate average AoI performance achieved at the receiver, meanwhile, increasing the ratio $\gamma_{d}/\gamma_{E}$ requires the transmitter to pay greater emission energy. All of these demonstrate that there are certain trade-offs among transmission security, transmission timeliness, and energy efficiency when sending packets on infinite capacity status updating systems.*

*Secondly, notice that Equations (13) and (15) represent average age gaps obtained by calculating positive and negative g’s. Non-ignorable negative part (15) indicates that it is necessary to take into consideration those negative g’s, i.e., negative security performance, when evaluating the transmission security of status updating systems. Therefore, previous works [40,41] who considered only the positive part where $g=n_{E}-n_{d}>0$, and designing optimal transmission policies based on positive security metrics may not truly achieve optimal performance. It sees from (13) and (15) that $\beta_{+}^{\prime }\left(1\right)$ and $\beta_{-}^{\prime }\left(1\right)$ are proportional to E[Y], thus when reducing packet generation rate to increase E[Y], both of positive and negetive parts of security metric are improved. Applying the general Formula (16) and infinite capacity system’s average AoI derived in previous work [36], transmission security and transmission timeliness will be jointly optimized in Section 4.*

*At last, it sees that the general expression of F, especially in Equations (17) and (18), demonstrate the symmetry of average age gap between eavesdropper and legitimate receiver with respect to $\gamma_{d}$ and $\gamma_{E}$. This symmetry is determined by the symmetrical position of two nodes. We speculate that this symmetry will still exist in other status updating systems, for example, in bufferless systems and finite capacity systems.*

### 3.2. Age Gap g in Bernoulli Arrival Process Case

In Section 3.1, assuming that *Y* follows an arbitrary distribution, we have derived the general Formula (16) for infinite capacity system’s freshness advantage. In special case of Bernoulli arrival process, the accurate distribution of instantaneous age gap *g* is determined in this subsection.

Let *Y* be a geometric random variable, that is,

(20) $$\mathrm{Pr}\left\{Y=j\right\}={\left(1-p\right)}^{j-1}p\;\;\left(j\geq 1\right)$$

where *p* is the probability that the source node generates a new packet in each time slot. In this case, we rewrite the stationary Equations (4) as follows.

(21) $$\left\{\begin{array}{cc} \pi_{g}=\pi_{g}\left(1-\gamma_{d}\right)\left(1-\gamma_{E}\right)+p\gamma_{d}\left(1-\gamma_{E}\right)\sum_{k=0}^{g-1}\pi_{k}{\left(1-p\right)}^{g-k-1} & \left(g\geq 1\right)\\ \pi_{0}=\pi_{0}\left[\left(1-\gamma_{d}\right)\left(1-\gamma_{E}\right)+\gamma_{d}\gamma_{E}\right]+\gamma_{d}\sum_{j=-\infty }^{-1}\pi_{j}+\gamma_{d}\gamma_{E}\sum_{k=1}^{+\infty }\pi_{k}\\ \pi_{g}=\pi_{g}\left(1-\gamma_{d}\right)+p\left(1-\gamma_{d}\right)\gamma_{E}\left(\sum_{k=0}^{+\infty }\pi_{k}\right){\left(1-p\right)}^{|g|-1} & \left(g\leq -1\right) \end{array}\right.$$

All of stationary probabilities $\pi_{g}$, g∈Z are fully determined by solving Equations (21). We state the obtained results in following Theorem 2.

**Theorem** **2.** *Assuming that packet interarrival time Y follows the geometric distribution with arriving rate p. In this case, the instantaneous age gap $g=n_{E}-n_{d}$ is distributed according to following equations. For k≥1,*

(22) $$\mathrm{Pr}\left\{g=k\right\}=\frac{p\gamma_{d}^{2}\gamma_{E}\left(1-\gamma_{E}\right){\left(1-\frac{p\gamma_{E}}{1-\left(1-\gamma_{d}\right)\left(1-\gamma_{E}\right)}\right)}^{k-1}}{{\left[1-\left(1-\gamma_{d}\right)\left(1-\gamma_{E}\right)\right]}^{3}}$$

*and,*

(23) $$\mathrm{Pr}\left\{g=0\right\}=\frac{\gamma_{d}\gamma_{E}}{{\left[1-\left(1-\gamma_{d}\right)\left(1-\gamma_{E}\right)\right]}^{2}}$$

*In addition, for each k≤−1,*

(24) $$\mathrm{Pr}\left\{g=k\right\}=\frac{p\left(1-\gamma_{d}\right)\gamma_{E}{\left(1-p\right)}^{|k|-1}}{1-\left(1-\gamma_{d}\right)\left(1-\gamma_{E}\right)}$$

*Notice that Pr{g=0} is independent of Y, such that for any packet arrival processes, Pr{g=0} is always determined by Equation (23).*

**Proof.**  Directly from the last row of (21), we obtain that for g≤−1,

(25) $$\pi_{g}=\frac{p\left(1-\gamma_{d}\right)\gamma_{E}\left(\sum_{k=0}^{+\infty }\pi_{k}\right){\left(1-p\right)}^{|g|-1}}{\gamma_{d}}$$

In Equation (A12), we have obtained that

$$\sum_{k=0}^{+\infty }\pi_{k}=\frac{\gamma_{d}}{1-\left(1-\gamma_{d}\right)\left(1-\gamma_{E}\right)}$$

which relies only on $\gamma_{d}$ and $\gamma_{E}$, thus also holds for geometric *Y* case. Substituting (A12) into (25) gives that

(26) $$\pi_{g}=\frac{p\left(1-\gamma_{d}\right)\gamma_{E}{\left(1-p\right)}^{|g|-1}}{1-\left(1-\gamma_{d}\right)\left(1-\gamma_{E}\right)}\;\left(g\leq -1\right)$$

Similarly, notice that in (A13), we determine that

(27) $$\pi_{0}=\frac{\gamma_{d}\gamma_{E}}{{\left[1-\left(1-\gamma_{d}\right)\left(1-\gamma_{E}\right)\right]}^{2}}$$

This is true for all the cases where *Y* follows an arbitrary distribution. Finally, from the first row of (21), we have that for g≥1,

(28) $$\frac{\left[1-\left(1-\gamma_{d}\right)\left(1-\gamma_{E}\right)\right]}{p\gamma_{d}\left(1-\gamma_{E}\right)}\pi_{g}=\sum_{k=0}^{g-1}\pi_{k}{\left(1-p\right)}^{g-k-1}$$

Do once interation yields

(29) $$\frac{\left[1-\left(1-\gamma_{d}\right)\left(1-\gamma_{E}\right)\right]}{p\gamma_{d}\left(1-\gamma_{E}\right)}\pi_{g-1}=\sum_{k=0}^{g-2}\pi_{k}{\left(1-p\right)}^{g-k-2}$$

It is easy to calculate that

(30) $$\frac{\left[1-\left(1-\gamma_{d}\right)\left(1-\gamma_{E}\right)\right]}{p\gamma_{d}\left(1-\gamma_{E}\right)}\left(\pi_{g}-\left(1-p\right)\pi_{g-1}\right)=\pi_{g-1}$$

from which we obtain

(31) $$\pi_{g}=\left(1-\frac{p\gamma_{E}}{1-\left(1-\gamma_{d}\right)\left(1-\gamma_{E}\right)}\right)\pi_{g-1}$$

Using Equation (31) repeatedly, it shows that

(32) $$\pi_{g}={\left(1-\frac{p\gamma_{E}}{1-\left(1-\gamma_{d}\right)\left(1-\gamma_{E}\right)}\right)}^{g-1}\pi_{1}$$

While letting g=1 in the first row of (21), we have

(33) $$\begin{array}{cc} \pi_{1} & =\frac{p\gamma_{d}\left(1-\gamma_{E}\right)}{1-\left(1-\gamma_{d}\right)\left(1-\gamma_{E}\right)}\pi_{0} \end{array}$$

(34) $$\begin{array}{cc} \; & =\frac{p\gamma_{d}^{2}\gamma_{E}\left(1-\gamma_{E}\right)}{{\left[1-\left(1-\gamma_{d}\right)\left(1-\gamma_{E}\right)\right]}^{3}} \end{array}$$

Substituting (34) into (32), we determine that for g≥1,

(35) $$\pi_{g}=\frac{p\gamma_{d}^{2}\gamma_{E}\left(1-\gamma_{E}\right){\left(1-\frac{p\gamma_{E}}{1-\left(1-\gamma_{d}\right)\left(1-\gamma_{E}\right)}\right)}^{g-1}}{{\left[1-\left(1-\gamma_{d}\right)\left(1-\gamma_{E}\right)\right]}^{3}}$$

Finally, collecting Equations (26), (27) and (35), we have fully determined the distribution of age gap *g*. This completes the proof of Theorem 2. □

Since

(36) $$\frac{\gamma_{E}}{1-\left(1-\gamma_{d}\right)\left(1-\gamma_{E}\right)}=\frac{\gamma_{E}}{\gamma_{E}+\gamma_{d}\left(1-\gamma_{E}\right)}<1$$

then we have that

(37) $$1-\frac{p\gamma_{E}}{1-\left(1-\gamma_{d}\right)\left(1-\gamma_{E}\right)}>1-p$$

Equations (22) and (24) show that as |g| increases, negative age gap *g* decays faster than positive *g*. We will depict this distribution in numerical simulation section.

## 4. Joint Optimization of Transmission Timeliness and Security for Infinite Capacity Systems

In this Section, we discuss the issue of jointly designing transmission timeliness and transmission security when sending packets through infinite capacity systems. The timeliness is characterized by average AoI ${\bar{\Delta }}_{d}$, which has been obtained in [36], while the transmission security is represented by freshness advantage *F*. We will consider following two problems. Firstly, in Section 4.1, for some α>0, we construct $Q={\bar{\Delta }}_{d}-\alpha F$ as combined performance comprising both timeliness and security metric, and determine the optimal packet generation rate $p^{*}$ such that *Q* is minimized. Secondly, under the conditions that ${\bar{\Delta }}_{d}$ and *F* are limited, in Section 4.2 the feasible region of $\left(p,\gamma_{d}\right)$ pairs are determined explicitly through numerical simulations, where we analyze and explain how these constrains affect the region composed of feasible *p*’s and $\gamma_{d}$’s.

### 4.1. Minimizing Combined Performance Metric Q

For Bernoulli packet arrivals, in work [36] we derived that average AoI of an infinite capacity system, ${\bar{\Delta }}_{d}$, is equal to

(38) $${\bar{\Delta }}_{d}=\frac{1}{\gamma_{d}}\left(\left(1-\gamma_{d}\right)+\frac{1}{\rho_{d}}+\frac{\rho_{d}^{2}\left(1-\gamma_{d}\right)}{1-\rho_{d}}\right)$$

in which $\rho_{d}=p/\gamma_{d}$ is discrete traffic intensity.

In order to optimize timeliness and security of transmitting packets simultaneously, we construct

$$\begin{array}{cc} Q & ={\bar{\Delta }}_{d}-\alpha F \end{array}$$

(39) $$\begin{array}{cc} \; & =\frac{1}{\gamma_{d}}\left(\left(1-\gamma_{d}\right)+\frac{1}{\rho_{d}}+\frac{\rho_{d}^{2}\left(1-\gamma_{d}\right)}{1-\rho_{d}}\right)-\frac{\alpha }{\rho_{d}\gamma_{d}}\left(\frac{\gamma_{d}}{\gamma_{E}}-1\right) \end{array}$$

(40) $$\begin{array}{cc} \; & =\frac{\left(1-\gamma_{d}\right)+\left[1-\alpha \left(\frac{\gamma_{d}}{\gamma_{E}}-1\right)\right]\frac{1}{\rho_{d}}+\frac{\rho_{d}^{2}\left(1-\gamma_{d}\right)}{1-\rho_{d}}}{\gamma_{d}} \end{array}$$

as weighted difference between average AoI ${\bar{\Delta }}_{d}$ and freshness advantage *F*, because we want to minimize average AoI and maximize freshness advantage, and indicate unequal emphasis levels on timeliness performance and security performance. In Equation (39), notice that $E\left[Y\right]=1/p=1/\left(\rho_{d}\gamma_{d}\right)$ in the case of Bernoulli packet arrivals.

**Theorem** **3.** *For given $\gamma_{d}$ and $\gamma_{E}$, the optimal packet generation rate $p^{*}$ that minimizes combined performance metric Q is determined as follows. If $\frac{1-\alpha (\gamma_{d}/\gamma_{E}-1)}{1-\gamma_{d}}\leq 0$, it shows that Q is increasing as p becomes large. Thus, $p^{*}$ is equal to some minimal allowable packet generation rate $p_{\min}$. While for the cases of $\frac{1-\alpha (\gamma_{d}/\gamma_{E}-1)}{1-\gamma_{d}}>0$, $p^{*}$ is determined to be*

(41) $$p^{*}=\rho_{d}^{*}\gamma_{d}$$

*in which $0<\rho_{d}^{*}<1$ is the real root of equation*

(42) $${\left(\frac{\rho_{d}}{1-\rho_{d}}\right)}^{2}-\rho_{d}^{2}=\frac{1-\alpha (\gamma_{d}/\gamma_{E}-1)}{1-\gamma_{d}}$$

**Proof.**  Considering *Q* as the function of $\rho_{d}$, then minimizing *Q* is the same as minimizing the numerator of (40), which is denoted as $Q_{n}$. Calculating following derivative and making it greater than zero, we obtain that

(43) $$\begin{array}{cc} Q_{n}^{\prime }\left(\rho_{d}\right) & =-\left[1-\alpha \left(\frac{\gamma_{d}}{\gamma_{E}}-1\right)\right]\frac{1}{\rho_{d}^{2}}+\frac{\left(1-\gamma_{d}\right)\left[2\rho_{d}\left(1-\rho_{d}\right)+\rho_{d}^{2}\right]}{{\left(1-\rho_{d}\right)}^{2}}\geq 0 \end{array}$$

which is equivalent to

(44) $$\begin{array}{c} \frac{\left(1-\gamma_{d}\right)\left[1-{\left(1-\rho_{d}\right)}^{2}\right]}{{\left(1-\rho_{d}\right)}^{2}}\geq \frac{1-\alpha (\gamma_{d}/\gamma_{E}-1)}{\rho_{d}^{2}}\Rightarrow {\left(\frac{\rho_{d}}{1-\rho_{d}}\right)}^{2}-\rho_{d}^{2}\geq \frac{1-\alpha (\gamma_{d}/\gamma_{E}-1)}{1-\gamma_{d}} \end{array}$$

Notice that Equation (44) is a quartic inequality, from which it is difficult to obtain the accurate expression of $\rho_{d}$. Instead, we give following analysis.

Define function $f(\rho_{d})$ as

(45) $$f\left(\rho_{d}\right)={\left(\frac{\rho_{d}}{1-\rho_{d}}\right)}^{2}-\rho_{d}^{2}$$

and denote

(46) $$\Gamma =\frac{1-\alpha (\gamma_{d}/\gamma_{E}-1)}{1-\gamma_{d}}$$

we consider the intersection point of $f(\rho_{d})$’s graph and the line of y=Γ.

Calculating the derivative of $f(\rho_{d})$ or drawing its graph, it is easy to verify that $f(\rho_{d})$ is monotonically increasing as $\rho_{d}$ becomes large, and $f{\left(\rho_{d}\right)}_{\min}=f\left(0\right)=0$. Therefore, in the first case when Γ≤0, $Q_{n}^{\prime }\left(\rho_{d}\right)\geq 0$ always holds true. This implies that $Q_{n}$ is increasing in all the range of $0<\rho_{d}<1$, thus $Q_{n}$, also *Q*, is minimized by setting $\rho_{d}$ to be the minimal allowable value $\rho_{d,\min}$. We denote the corresponding packet generation rate to be $p_{\min}=\rho_{d,\min}\gamma_{d}$.

Consider the other case where Γ>0. Since $f(\rho_{d})$ is an increasing function from 0, thus for any Γ>0, there exists the unique $\rho_{d}^{*}\in \left(0,1\right)$ such that

(47) $$f\left(\rho_{d}^{*}\right)={\left(\frac{\rho_{d}^{*}}{1-\rho_{d}^{*}}\right)}^{2}-{\left(\rho_{d}^{*}\right)}^{2}=\Gamma$$

and in the range of $\left(0,\rho_{d}^{*}\right)$, $Q_{n}$, *Q* decreases, while for $\rho_{d}^{*}<\rho_{d}<1$, $Q_{n}$, *Q* increases. Thus, $p^{*}=\rho_{d}^{*}\gamma_{d}$ minimizes the combined metric *Q*. Since it is difficult to obtain the accurate expression of $\rho_{d}^{*}$, in following Table 2 we list the numerical correspondence between optimal traffic intensity $\rho_{d}^{*}$ and Γ.

So far, summarize above discussions, we complete the proof of Theorem 3. □

### 4.2. Feasible p and $\gamma_{d}$ When Timeliness and Security Are Limited

**Figure 2:**
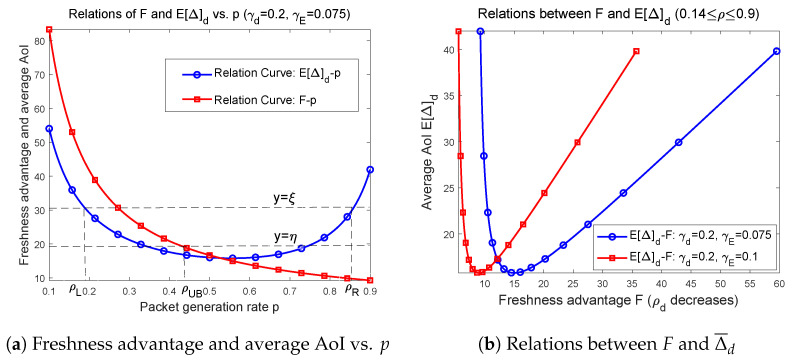
Relationships between freshness advantage *F*, average AoI ${\bar{\Delta }}_{d}$ and $\rho_{d}$, as well as their interrelations.

For some given ξ,η>0, and assuming that ${\bar{\Delta }}_{d}\leq \xi$, F≥η, in the second part of this Section, we describe the feasible region composed of *p* and $\gamma_{d}$. Notice that in designing transmission performance of status updating systems, both packet generation rate *p* and transmission success probability to legitimate receiver $\gamma_{d}$ are adjustable. We depict the situation that ${\bar{\Delta }}_{d}\leq \xi$, F≥η and mark some points in Figure 2a.

We consider the case where packet arrivals form a Bernoulli process. According to Equations (16) and (38), we require that

(48) $$\frac{1}{\gamma_{d}}\left(\left(1-\gamma_{d}\right)+\frac{\gamma_{d}}{p}+\frac{p^{2}\left(1-\gamma_{d}\right)}{\gamma_{d}\left(\gamma_{d}-p\right)}\right)\leq \xi$$

and

(49) $$\frac{1}{p}\left(\frac{\gamma_{d}}{\gamma_{E}}-1\right)\geq \eta$$

which represent transmission timeliness constraint and transmission security constraint.

From (49), it is easy to obtain that

(50) $$\gamma_{d}\geq p\gamma_{E}\eta +\gamma_{E}$$

That is, security requirement (49) derives a linear constraint between $\gamma_{d}$ and *p*. While timeliness requirement (48) provides a nonlinear constraint which is difficult to describe.

**Figure 3:**
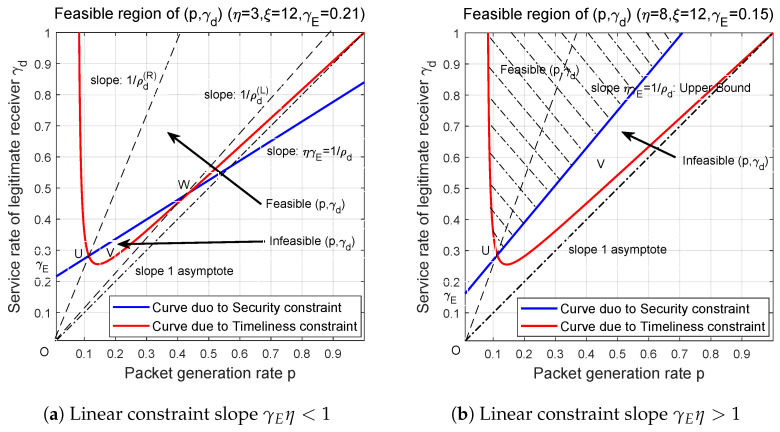
Feasible region of $\left(p,\gamma_{d}\right)$ pairs under the constraints that ${\bar{\Delta }}_{d}\leq \xi$, F≥η.

In the following, through numerical examples, we depict the feasible $\left(p,\gamma_{d}\right)$ pairs such that both of (48) and (49) are satisfied. According to the slope of linear constraint (50), i.e., $\gamma_{E}\eta$ is greater than 1 or less than 1, in Figure 3a,b, we describe the feasible regions in two cases.

In Figure 3a, the blue line represents the linear security constraint, and its intersection with the vertical axis is equal to $\gamma_{E}$, that is, the success probability of transmitting a packet to eavesdropper. While the red line shows the nonlinear constraint incurred by timeliness requirement. It is not hard to determine that the feasible $\left(p,\gamma_{d}\right)$ pairs are located above the blue and red lines. For the red line, when fixing packet generation rate *p*, the average AoI is monotonically decreasing as $\gamma_{d}$ increases. Therefore, when some $\gamma_{d}$ meets the constraint (48), any $\gamma_{d}^{\prime }>\gamma_{d}$ can also satisfy (48). This explains that all the feasible $\left(p,\gamma_{d}\right)$ are located above the red and blue line. Since the *y*-axis denotes $\gamma_{d}$ and the *x*-axis represents *p*, then the slope of any line drawn from the origin is equal to the reciprocal of traffic intensity, i.e., $1/\rho_{d}$. In order to ensure the stability of the service system (status updating system), we have $\rho_{d}<1$. Thus, the feasible *p* and $\gamma_{d}$ pairs are located above $\gamma_{d}=p$ line, and it sees that the red curve which represents timeliness constraint approaches the asymptote $\gamma_{d}=p$ when p→1. Figure 3a shows that the infeasible $\left(p,\gamma_{d}\right)$ pairs that do not meet security constraint (49) are located in the bottom of red line. Here, an interesting point is that when we consider the impact of transmission security constraints on traffic intensity $\rho_{d}$, it turns out that there is in fact no limitation on the feasible range of $\rho_{d}$. This is because for any point located in infeasible region, say $V(p,\gamma_{d})$, by connecting OV and extending the line, we can always find a point $V^{\prime }\left(p^{\prime },\gamma_{d}^{\prime }\right)$ in feasible region, such that

(51) $$\rho_{d}\left(V\right)=\frac{p}{\gamma_{d}}=\frac{p^{\prime }}{\gamma_{d}^{\prime }}=\rho_{d}\left(V^{\prime }\right)$$

From this example, we show that using feasible $\left(p,\gamma_{d}\right)$ region to describe the feasible system parameter settings are more accurate than using the single parameter $\rho_{d}$.

For $\gamma_{E}\eta >1$, we plot the blue line and the red curve that correspond to timeliness and security constraints in Figure 3b, in which the feasible region of $\left(p,\gamma_{d}\right)$’s are clearly marked with dashed lines. Notice that comparing with previous case where $\gamma_{E}\eta <1$, in this case the probability that a transmitted packet is eavesdropped becomes large. Similarly, the red curve has the asymptote $\gamma_{d}=p$ when *p* approaches to 1, and the feasible $\left(p,\gamma_{d}\right)$ pairs are located above the blue and red lines. Unlike the situation of $\gamma_{E}\eta <1$, notice that the infeasible $\left(p,\gamma_{d}\right)$’s form an open area. From the perspective of $\rho_{d}$, in this case not all $\rho_{d}$’s that meet the timeliness constraint can also meet the security constraint. Observing that for some infeasible pair $\left(p,\gamma_{d}\right)$, say point *V* in Figure 3b, connecting the segment OV and extending, it sees that all the lines are located within infeasible region, which means that when setting traffic intensity $\rho_{d}$ to be $p/\gamma_{d}$, transmission timeliness constraint is satisfied but transmission security constraint is violated. In other words, for the case of $\gamma_{E}\eta >1$, security constraint (49) truly limits the range of $\rho_{d}$’s, if we use $\rho_{d}$ to distinguish the feasible and infeasible cases. More specifically, Figure 3b indicates that in order to meet the security constraint, very high $\rho_{d}$’s that are represented by $\left(p,\gamma_{d}\right)$’s near the asymptote $\gamma_{d}=p$, are not feasible. Thus, the reciprocal of blue line’s slope, i.e., $1/(\eta \gamma_{E})$, approximately provides an upper bound of feasible $\rho_{d}$’s, especially when $\gamma_{E}$, i.e., the intersection of linear security constraint and the vertical axis, is small.

Finally, when reducing the timeliness threshold ξ and increasing the security threshold η, the red curve will move upward, and the blue line will rotate upward to the left with $\left(0,\gamma_{E}\right)$ as the endpoint. This will narrow down the feasible region of $\left(p,\gamma_{d}\right)$. On the contrary, by increasing ξ and reducing η, we can expand the feasible area, which means more $\left(p,\gamma_{d}\right)$ pairs can meet the timeliness and security constraints.

## 5. Numerical Simulations

In this Section, dividing into two parts, the numerical simulations for the main results obtained in the paper are provided. In Section 5.1, the relations between freshness advantage *F* and traffic intensity $\rho_{d}$ are depicted, where in the same figure average AoI of the system is also plotted. Then, in order to explicitly show the tradeoffs between *F* and average AoI ${\bar{\Delta }}_{d}$, we plot ${\bar{\Delta }}_{d}-F$ curves to show the direct relationships between ${\bar{\Delta }}_{d}$ and *F*. According to Theorem 2 and Table 2, in Section 5.2 we draw the distribution of instantaneous age gap *g* and illustrate the correspondence between the optimal traffic intensity $\rho_{d}^{*}$ and the given Γ, which is defined in (46).

### 5.1. Relationship Between F, ${\bar{\Delta }}_{d}$ and $\rho_{d}$ as Well as Their Interrelations

In Figure 2a, we simultaneously depict the changing curves of freshness advantage *F* and average AoI ${\bar{\Delta }}_{d}$ as $\rho_{d}$ increases. It has been proved previously that average AoI of an infinite capacity status updating system first decreases then increases when traffic intensity ρ becomes large, which indicates the tradeoffs between server’s utilization and network congestion. From the general formula of freshness advantage *F* in Equation (16), it sees that *F* will decrease monotonically with $\rho_{d}$. Notice that *F* is also related to the eavesdropping probability $\gamma_{E}$. When reducing $\gamma_{E}$, that is decreasing the probability that the eavesdropper obtains packets, then *F* increases and the corresponding $F-\rho_{d}$ curve moves upward. More specifically, in Figure 2a, as $\rho_{d}$ increases from 0.55 to 0.9, it shows that freshness advantage *F* decreases while average AoI${\bar{\Delta }}_{d}$ increases, representing that both of transmission timeliness and transmission security of the system are deteriorating, which is the least desirable situation. On the other hand, when $\rho_{d}$ varies from 0.1 to 0.55, Figure 2a shows that average AoI decreases but freshness advantage *F* also decreases, from which we can conclude that the enhancement of system’s timeliness performance comes at the cost of the weakening of system’s security performance.

In order to clearly reveal the relationships between transmission timeliness and transmission security for an infinite capacity status updating system, in Figure 2b we provide the ${\bar{\Delta }}_{d}-F$ curves when setting different system parameters. These graphs are depicted by representing ${\bar{\Delta }}_{d}$ and *F* to be parameter equations of $\rho_{d}$. Figure 2b shows that as *F* increases (in this case, $\rho_{d}$ is decreasing), average AoI of the system is first decreasing, reaching the minimum value, and then increasing. For the cases where the security constraint is not strict, i.e., *F* is not required to be very large, for example, not exceeding 20 in Figure 2b, a low level average AoI can be obtained and at the same time meeting the security limitation. However, if it requires that freshness advantage *F* must be greater than some given large threshold, then Figure 2b clearly shows that system’s average AoI cannot take small values. This implies that if we want good transmission security, we must accept the compromise on transmission timeliness. From the perspective of freshness advantage *F* and average AoI ${\bar{\Delta }}_{d}$, based on numerical simulations, we point out that there is no optimal system designs that can optimize both *F* and ${\bar{\Delta }}_{d}$ at the same time.

### 5.2. Distribution of Instantaneous Gap g and the Correspondence Between Γ and $p^{*}$

**Figure 4:**
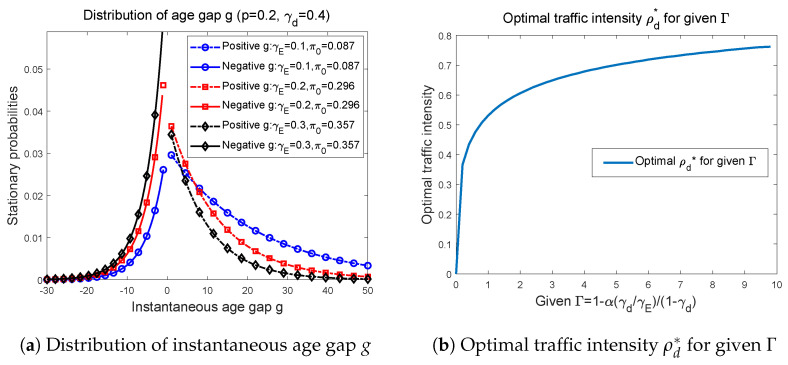
Distribution of instantaneous age gap *g* and the optimal traffic intensity $\rho_{d}^{*}$ corresponding to given Γ.

In Figure 4a, by fixing $p=0.2,\gamma_{d}=0.4$ and setting $\gamma_{E}$ to be 0.1, 0.2, and 0.3, we plot the probability distribution of instantaneous age gap *g*. Strictly speaking, because the age age *g* takes only integer values, we should draw a series of scatter points. To better illustrate distributional trend of *g*, in Figure 4a we have plotted two continuous curves, one for positive *g*s and the other for negative *g*s. Notice that the distribution curves of negative *g*’s and positive *g*’s are depicted separately, and we point out that the probability that *g* equals 0 is discontinuous with the distributions on both sides, therefore, we list $\pi_{0}$ individually in the legend of the figure. It sees that the distribution of *g* is centered around 0 and decays towards both sides. As we pointed out in brief discussion below Theorem 2’s proof, as |g| increases, the distribution curve of negative *g*’s has a greater decay rate than that of positive *g*’s. Careful observations show that for negative *g*, when increasing $\gamma_{E}$, the probability of taking the same *g* is also increasing, and the distribution curves decreases faster as $\gamma_{E}$ increases. On the contrary, for the positive side, the decay rate of the curve corresponding to smaller $\gamma_{E}$ is slower. For slightly large *g*’s, the situations that probabilities distribute are opposite to the cases of negative *g*’s. More specifically, for some g<0, it shows that $\pi_{g}\left(0.1\right)<\pi_{g}\left(0.2\right)<\pi_{g}\left(0.3\right)$. However, on the positive side, we have $\pi_{-g}\left(0.3\right)<\pi_{-g}\left(0.2\right)<\pi_{-g}\left(0.1\right)$. This is because the probability distributions corresponding to same color curves in the positive and negative parts are complementary. In particular, for case of g=0, we show that for all of three cases depicted in Figure 4a, $\pi_{0}$ is much higher than the probabilities that *g* takes other values. Overall, the numerical simulation of *g*’s distribution shows the symmetrical and decaying trend, which actually demonstrate the symmetry of instantaneous age gap $g=n_{E}-n_{d}$, because we can treat the eavesdropper as another legitimate receiver.

At last, in Figure 4b we draw the correspondence relationships between optimal traffic intensity $\rho_{d}^{*}$ and the given Γ, where we let Γ varies from 0 to 10. Numerical result verifies that as Γ increases, $\rho_{d}^{*}$ is increasing with a decreasing rate. Generally speaking, it shows that the optimal traffic intensity $\rho_{d}^{*}$, also the optimal packet generation rate $p^{*}$, is the concave function of Γ. Here, we provide following discussions about the values that Γ takes.

Remember than we define in Equation (46) that

(52) $$\Gamma =\frac{1-\alpha (\gamma_{d}/\gamma_{E}-1)}{1-\gamma_{d}}$$

in which α>0. Assuming that $\gamma_{d}/\gamma_{E}>1$, then $\alpha (\gamma_{d}/\gamma_{E}-1)>0$, which gives that

(53) $$\Gamma =\frac{1-\alpha (\gamma_{d}/\gamma_{E}-1)}{1-\gamma_{d}}<\frac{1}{1-\gamma_{d}}$$

i.e., Γ is upper bounded. While for $\gamma_{d}/\gamma_{E}<1$, since α>0, it is easy to obtain that

(54) $$\Gamma =\frac{1-\alpha (\gamma_{d}/\gamma_{E}-1)}{1-\gamma_{d}}>\frac{1}{1-\gamma_{d}}$$

is lower bounded. Therefore, for example, if setting $\gamma_{d}=0.8$, then in Figure 4b, 0<Γ<5 corresponds to the cases where $\gamma_{d}>\gamma_{E}$, and Γ>5 only occurs when $\gamma_{d}<\gamma_{E}$.

## 6. Conclusions

In this paper, assuming that there exist passive eavesdroppers, we assess the security performance of timely transmitting packets on infinite capacity status updating systems. We define freshness advantage *F* as transmission security metric, which is equal to average instantaneous gap between eavesdropper’s AoI and legitimate receiver’s AoI. We derive the general formula of *F* and prove that *F* depends only on average packet interarrival time and the ratio $\gamma_{d}/\gamma_{E}$. For special case of Bernoulli(p) packet arrivals, the explicit distribution of age gap $g=n_{E}-n_{d}$ is calculated. Notice that the eavesdropper can be treated as another legitimate receiver, we demonstrate the symmetry in freshness advantage *F* and its probability distribution. This is a point that was not mentioned in previous works. To design and optimize system’s average AoI${\bar{\Delta }}_{d}$ and freshness advantage *F* simultaneously, on the one hand, we construct the combined performance *Q* and determine the optimal *p* that minimizes it. On the other hand, by imposing constraints on ${\bar{\Delta }}_{d}$ and *F*, the feasible regions of *p* and $\gamma_{d}$ are determined through numerical examples. Finally, we provide sufficient numerical simulations to explain the relationships between *F* and other system parameters, as well as the relations between *F* and average AoI${\bar{\Delta }}_{d}$, so that revealing the tradeoffs between transmission timeliness and transmission security. It proves that there is no system designs that can optimize timeliness and security at the same time.

For possible future works, one direction is deriving the freshness advantage *F* for bufferless and finite capacity status updating systems, so that characterizing the transmission security of these systems. Compared to previous works, allowing age gap *g* to take arbitrary values will lead to more comprehensive results. Another major problem of this paper is that we do not deal with the packet when it is eavesdropped and becomes insecure. Notice that this will cause information leakage at the receiver side. We point out that how to handle insecure packets and determine the accurate tradeoffs between the amount of leaked information and the average AoI will be very meaningful and valuable topics in future research works.

## Figures and Tables

**Table 1:** Random Transfers of Instantaneous Age Gap $g=\gamma_{E}-\gamma_{d}$.

Initial State	R.V.s	Next State and Transtion Probability
g>0, $n_{E}>n_{d}$	$B_{d},B_{E}$	(0,0): *g* with $\left(1-\gamma_{d}\right)\left(1-\gamma_{E}\right)$
		(0,1): −y with $\left(1-\gamma_{d}\right)\gamma_{E}\mathrm{Pr}\left\{Y=y\right\}$
		(1,0): g+y with $\gamma_{d}\left(1-\gamma_{E}\right)\mathrm{Pr}\left\{Y=y\right\}$
		(1,1): 0 with $\gamma_{d}\gamma_{E}$
g=0, $n_{E}=n_{d}$	$B_{d},B_{E}$	(0,0): 0 with $\left(1-\gamma_{d}\right)\left(1-\gamma_{E}\right)$
		(0,1): −y with $\left(1-\gamma_{d}\right)\gamma_{E}\mathrm{Pr}\left\{Y=y\right\}$
		(1,0): *y* with $\gamma_{d}\left(1-\gamma_{E}\right)\mathrm{Pr}\left\{Y=y\right\}$
		(1,1): 0 with $\gamma_{d}\gamma_{E}$
g<0, $n_{E}<n_{d}$	$B_{d}$	$B_{d}=0$: *g* with $1-\gamma_{d}$
		$B_{d}=1$: 0 with $\gamma_{d}$

**Table 2:** Correspondence Between Optimal Traffic Intensity $\rho_{d}^{*}$ and Γ.

Mathematic Relation	Γ	$\rho_{d}^{*}$	Γ	$\rho_{d}^{*}$	Γ	$\rho_{d}^{*}$	Γ	$\rho_{d}^{*}$	Γ	$\rho_{d}^{*}$
${\left(\frac{\rho_{d}^{*}}{1-\rho_{d}^{*}}\right)}^{2}-{\left(\rho_{d}^{*}\right)}^{2}=\Gamma$, $\Gamma =\frac{1-\alpha (\gamma_{d}/\gamma_{E}-1)}{1-\gamma_{d}}$	0	0	2.0	0.606	4.0	0.679	6.0	0.719	8.0	0.745
	0.2	0.366	2.2	0.616	4.2	0.684	6.2	0.722	8.2	0.747
	0.4	0.434	2.4	0.626	4.4	0.688	6.4	0.725	8.4	0.750
	0.6	0.476	2.6	0.634	4.6	0.693	6.6	0.728	8.6	0.752
	0.8	0.507	2.8	0.642	4.8	0.697	6.8	0.730	8.8	0.754
	1.0	0.531	3.0	0.649	5.0	0.701	7.0	0.733	9.0	0.756
	1.2	0.551	3.2	0.656	5.2	0.705	7.2	0.736	9.2	0.758
	1.4	0.568	3.4	0.662	5.4	0.708	7.4	0.738	9.4	0.760
	1.6	0.582	3.6	0.668	5.6	0.712	7.6	0.741	9.6	0.761
	1.8	0.595	3.8	0.673	5.8	0.715	7.8	0.743	9.8	0.762

## Data Availability

Data are contained within the article.

## Appendix A. Proof of Lemma 1

In this appendix, $\beta_{+}\left(z\right)$, $\beta_{-}\left(z\right)$ are determined using Equations (4).

Firstly, merging the first two rows, we show that for g≥1,

(A1) $$\begin{array}{cc} \pi_{g} & =\pi_{g}\left(1-\gamma_{d}\right)\left(1-\gamma_{E}\right)+\gamma_{d}\left(1-\gamma_{E}\right)\left(\sum_{k=0}^{g-1}\pi_{k}\mathrm{Pr}\left\{Y=g-k\right\}\right) \end{array}$$

Then, $\beta_{+}\left(z\right)$ is calculated as

$$\begin{array}{cc} \beta_{+}\left(z\right) & =\sum_{g=1}^{+\infty }z^{g}\pi_{g}\\ \; & =\sum_{g=1}^{+\infty }z^{g}\left[\pi_{g}\left(1-\gamma_{d}\right)\left(1-\gamma_{E}\right)+\gamma_{d}\left(1-\gamma_{E}\right)\left(\sum_{k=0}^{g-1}\pi_{k}\mathrm{Pr}\left\{Y=g-k\right\}\right)\right] \end{array}$$

(A2) $$\begin{array}{cc} \; & =\left(1-\gamma_{d}\right)\left(1-\gamma_{E}\right)\left(\sum_{g=1}^{+\infty }z^{g}\pi_{g}\right)+\gamma_{d}\left(1-\gamma_{E}\right)\left(\pi_{0}+\sum_{g=1}^{+\infty }z^{g}\pi_{g}\right)Y\left(z\right) \end{array}$$

(A3) $$\begin{array}{cc} \; & =\left(1-\gamma_{d}\right)\left(1-\gamma_{E}\right)\beta_{+}\left(z\right)+\gamma_{d}\left(1-\gamma_{E}\right)\left(\pi_{0}+\beta_{+}\left(z\right)\right)Y\left(z\right) \end{array}$$

in which

(A4) $$\sum_{g=1}^{+\infty }z^{g}\left(\sum_{k=0}^{g-1}\pi_{k}\mathrm{Pr}\left\{Y=g-k\right\}\right)=\left(\pi_{0}+\sum_{g=1}^{+\infty }z^{g}\pi_{g}\right)Y\left(z\right)$$

is obtained by exchanging the summation order, and

(A5) $$Y\left(z\right)=\sum_{j=1}^{+\infty }z^{j}\mathrm{Pr}\left\{Y=j\right\}$$

is the probability generation function (PGF) of *Y*, i.e., random packet interarrival time.

From Equation (A3) we solve that

(A6) $$\beta_{+}\left(z\right)=\frac{\pi_{0}\gamma_{d}\left(1-\gamma_{E}\right)Y\left(z\right)}{1-\left(1-\gamma_{d}\right)\left(1-\gamma_{E}\right)-\gamma_{d}\left(1-\gamma_{E}\right)Y\left(z\right)}$$

Similarly, we first write the last row of (4) as

(A7) $$\pi_{g}=\pi_{g}\left(1-\gamma_{d}\right)+\left(1-\gamma_{d}\right)\gamma_{E}\left(\sum_{k=0}^{+\infty }\pi_{k}\right)\mathrm{Pr}\left\{Y=|g|\right\}$$

then calculate $\beta_{-}\left(z\right)$ as

$$\begin{array}{cc} \beta_{-}\left(z\right) & =\sum_{g=-\infty }^{-1}z^{\left|g\right|}\pi_{g}\\ \; & =\sum_{g=-\infty }^{-1}z^{\left|g\right|}\left[\pi_{g}\left(1-\gamma_{d}\right)+\left(1-\gamma_{d}\right)\gamma_{E}\left(\sum_{k=0}^{+\infty }\pi_{k}\right)\mathrm{Pr}\left\{Y=|g|\right\}\right]\\ \; & =\left(1-\gamma_{d}\right)\beta_{-}\left(z\right)+\left(1-\gamma_{d}\right)\gamma_{E}\left(\sum_{k=0}^{+\infty }\pi_{k}\right)Y\left(z\right) \end{array}$$

from which we obtain

$$\beta_{-}\left(z\right)=\frac{\left(1-\gamma_{d}\right)\gamma_{E}\left(\sum_{k=0}^{+\infty }\pi_{k}\right)Y\left(z\right)}{\gamma_{d}}$$

(A8) $$=\frac{\left(1-\gamma_{d}\right)\gamma_{E}\left(\pi_{0}+\beta_{+}\left(1\right)\right)Y\left(z\right)}{\gamma_{d}}$$

To derive the complete expressions of $\beta_{+}\left(z\right)$, $\beta_{-}\left(z\right)$, in following part we determine $\pi_{0}$ and $\pi_{0}+\beta_{+}\left(1\right)$.

In Equation (A6), letting z=1 gives that

(A9) $$\beta_{+}\left(1\right)=\frac{\pi_{0}\gamma_{d}\left(1-\gamma_{E}\right)}{\gamma_{E}}$$

In addition, since all of stationary probabilities add up to 1, we have

$$\begin{array}{cc} 1 & =\sum_{g=-\infty }^{-1}\pi_{g}+\pi_{0}+\sum_{g=1}^{+\infty }\pi_{g}\\ \; & =\beta_{-}\left(1\right)+\pi_{0}+\beta_{+}\left(1\right) \end{array}$$

(A10) $$\begin{array}{cc} \; & =\frac{\left(1-\gamma_{d}\right)\gamma_{E}\left(\pi_{0}+\beta_{+}\left(1\right)\right)}{\gamma_{d}}+\left(\pi_{0}+\beta_{+}\left(1\right)\right) \end{array}$$

(A11) $$\begin{array}{cc} \; & =\frac{\left[1-\left(1-\gamma_{d}\right)\left(1-\gamma_{E}\right)\right]\left(\pi_{0}+\beta_{+}\left(1\right)\right)}{\gamma_{d}} \end{array}$$

where Equation (A8) is used to obtain (A10). Equation (A11) gives that

(A12) $$\pi_{0}+\beta_{+}\left(1\right)=\frac{\gamma_{d}}{1-\left(1-\gamma_{d}\right)\left(1-\gamma_{E}\right)}$$

Combining (A9) and (A12), one can solve that

(A13) $$\pi_{0}=\frac{\gamma_{d}\gamma_{E}}{{\left[1-\left(1-\gamma_{d}\right)\left(1-\gamma_{E}\right)\right]}^{2}}$$

Substituting (A12) into Equation (A8), we determine that

(A14) $$\beta_{-}\left(z\right)=\frac{\left(1-\gamma_{d}\right)\gamma_{E}Y\left(z\right)}{1-\left(1-\gamma_{d}\right)\left(1-\gamma_{E}\right)}$$

Finally, collecting Equations (A6), (A13) and (A14), we complete the proof of Lemma 1.

## References

[1] Kaul S., Gruteser M., Rai V., Kenney J. Minimizing age of information in vehicular networks. Proceedings of the 2011 8th Annual IEEE Communications Society Conference on Sensor, Mesh and Ad Hoc Communications and Networks (SECOM).

[2] Kaul S., Yates R., Gruteser M. Real-time status: How often should one update?. Proceedings of the IEEE Computer and Communications Societies (INFOCOM).

[3] Kaul S.K., Yates R.D., Gruteser M. Status updates through queues. Proceedings of the 2012 46th Annual Conference on Information Sciences and Systems (CISS).

[4] Sun Y., Uysal-Biyikoglu E., Yates R., Koksal C.E., Shroff N.B. Update or wait: How to keep your data fresh. Proceedings of the 35th Annual IEEE International Conference on Computer Communications.

[5] Inoue Y., Masuyama H., Takine T., Tanaka T. (2019). A General Formula for the Stationary Distribution of the Age of Information and Its Application to Single-Server Queues. IEEE Trans. Inf. Theory.

[6] Yates R.D., Kaul S.K. (2019). The Age of Information: Real-Time Status Updating by Multiple Sources. IEEE Trans. Inf. Theory.

[7] Pappas N., Gunnarsson J., Kratz L., Kountouris M., Angelakis V. Age of information of multiple sources with queue management. Proceedings of the 2015 IEEE International Conference on Communications (ICC).

[8] Kosta A., Pappas N., Ephremides A., Angelakis V. (2019). Age of information performance of multiaccess strategies with packet management. J. Commun. Netw..

[9] Kaul S.K., Yates R.D. Age of Information: Updates with Priority. Proceedings of the IEEE International Symposium on Information Theory (ISIT).

[10] Xu J., Gautam N. (2021). Peak Age of Information in Priority Queuing Systems. IEEE Trans. Inf. Theory.

[11] Dogan O., Akar N. (2021). The Multi-Source Probabilistically Preemptive M/PH/1/1 Queue with Packet Errors. IEEE Trans. Commun..

[12] Kam C., Kompella S., Nguyen G.D., Wieselthier J.E., Ephremides A. Age of information with a packet deadline. Proceedings of the IEEE International Symposium on Information Theory (ISIT).

[13] Kam C., Kompella S., Nguyen G.D., Wieselthier J.E., Ephremides A. (2018). On the Age of Information with Packet Deadlines. IEEE Trans. Inf. Theory.

[14] Inoue Y. Analysis of the Age of Information with Packet Deadline and Infinite Buffer Capacity. Proceedings of the IEEE International Symposium on Information Theory (ISIT).

[15] Kam C., Kompella S., Nguyen G.D., Ephremides A. (2016). Effect of Message Transmission Path Diversity on Status Age. IEEE Trans. Inf. Theory.

[16] Javani A., Zorgui M., Wang Z. (2024). Age of Information for Multiple-Source Multiple-Server Networks. IEEE-ACM Trans. Netw..

[17] Chen Z., Yang T., Pappas N., Yang H.H., Tian Z., Wang M. (2024). Improving Information Freshness via Multi-Sensor Parallel Status Updating. IEEE Trans. Commun..

[18] Yates R.D. The Age of Gossip in Networks. Proceedings of the 2021 IEEE International Symposium on Information Theory (ISIT).

[19] Yates R.D. Timely Gossip. Proceedings of the 2021 IEEE 22nd International Workshop on Signal Processing Advances in Wireless Communications (SPAWC).

[20] Kaswan P., Bastopcu M., Ulukus S., Etesami S.R., Basar T. Optimizing Profitability in Timely Gossip Networks. Proceedings of the 2024 IEEE 25th International Workshop on Signal Processing Advances in Wireless Communications (SPAWC).

[21] Buyukates B., Bastopcu M., Ulukus S. Age of Gossip in Networks with Community Structure. Proceedings of the 2021 IEEE 22nd International Workshop on Signal Processing Advances in Wireless Communications (SPAWC).

[22] Bastopcu M., Buyukates B., Ulukus S. Gossiping with Binary Freshness Metric. Proceedings of the 2021 IEEE Globecom Workshops (GC Wkshps).

[23] Buyukates B., Bastopcu M., Ulukus S. (2022). Version Age of Information in Clustered Gossip Networks. IEEE J. Sel. Areas Inf. Theory.

[24] Ramakanth R.V., Tripathi V., Modiano E. (2025). Monitoring Correlated Sources: AoI-Based Scheduling is Nearly Optimal. IEEE Trans. Mob. Comput..

[25] Moradian M., Dadlani A., Khonsari A., Tabassum H. (2024). Age-Aware Dynamic Frame Slotted ALOHA for Machine-Type Communications. IEEE Trans. Commun..

[26] Tang Z., Yang N., Sadeghi P., Zhou X. (2023). Age of Information in Downlink Systems: Broadcast or Unicast Transmission?. IEEE J. Sel. Areas Commun..

[27] Wang Q., Chen H. (2023). Age of Information in Reservation Multi-Access Networks With Stochastic Arrivals: Analysis and Optimization. IEEE Trans. Commun..

[28] Fountoulakis E., Charalambous T., Ephremides A., Pappas N. (2023). Scheduling Policies for AoI Minimization with Timely Throughput Constraints. IEEE Trans. Commun..

[29] Zheng H., Xiong K., Fan P., Zhong Z., Letaief K.B. (2021). Age of Information-Based Wireless Powered Communication Networks with Selfish Charging Nodes. IEEE J. Sel. Areas Commun..

[30] Feng S., Yang J. (2022). Precoding and Scheduling for AoI Minimization in MIMO Broadcast Channels. IEEE Trans. Inf. Theory.

[31] Tripathi V., Talak R., Modiano E. (2019). Age of Information for Discrete Time Queues. arXiv.

[32] Kosta A., Pappas N., Ephremides A., Angelakis V. Non-linear Age of Information in a Discrete Time Queue: Stationary Distribution and Average Performance Analysis. Proceedings of the IEEE International Conference on Communications (ICC).

[33] Kosta A., Pappas N., Ephremides A., Angelakis V. (2021). The Age of Information in a Discrete Time Queue: Stationary Distribution and Non-Linear Age Mean Analysis. IEEE J. Sel. Areas Commun..

[34] Akar N., Dogan O. (2021). Discrete-Time Queueing Model of Age of Information with Multiple Information Sources. IEEE Internet Things J..

[35] Zhang J., Xu Y. On Age of Information for Discrete Time Status Updating System with Ber/G/1/1 Queues. Proceedings of the IEEE Information Theory Workshop (ITW).

[36] Zhang J., Xu Y. On Age of Information for Discrete Time Status Updating System with Infinite Size. Proceedings of the IEEE Information Theory Workshop (ITW).

[37] Zhang J., Xu Y. (2022). Age Analysis of Status Updating System with Probabilistic Packet Preemption. Entropy.

[38] Zhang J., Xu H., Cao D., Xu Y. (2024). Discrete Age of Information for Bufferless System with Multiple Prioritized Sources. IEEE Internet Things J..

[39] Yang Y., Hanzo L. (2023). Permutation-Based Short-Packet Transmissions Improve Secure URLLCs in the Internet of Things. IEEE Internet Things J..

[40] Yang Y. Secure and Timely Status Updates in the IoT using Short-Packet Permutation-Based Transmissions. Proceedings of the 2023 IEEE 98th Vehicular Technology Conference (VTC2023-Fall).

[41] Wang Q., Chen H., Mohapatra P., Pappas N. Secure Status Updates under Eavesdropping: Age of Information-based Secrecy Metrics. Proceedings of the IEEE Conference on Computer Communications Workshops (INFOCOM WKSHPS).

[42] Xu H., Hu Y., Zhu Y., Yuan X., Schmeink A. (2024). Achieving Secure and Fresh Information Updates via Short-Packet Communications. IEEE Wirel. Commun. Lett..

[43] Yuan F., Tang S., Liu D. (2024). AoI-Based Transmission Scheduling for Cyber Physical Systems Over Fading Channel Against Eavesdropping. IEEE Internet Things J..

[44] Wyner A.D. (1975). The wiretap channel. Bell Syst. Tech. J..

[45] Ma Y., Liu K., Liu Y., Zhu L. (2024). Timeliness and Secrecy-Aware Uplink Data Aggregation for Large-Scale UAV-IoT Networks. IEEE Internet Things J..

[46] Costa M., Sagduyu Y.E. Timely NextG Communications with Decoy Assistance against Deep Learning-based Jamming. Proceedings of the IEEE International Conference on Communications Workshops (ICC Workshops).

[47] Kim D., Yun S., Lee S., Lee J., Quek T.Q.S. (2024). Reinforcement Learning-Based Sensing Decision for Data Freshness in Blockchain-Empowered Wireless Networks. IEEE Wirel. Commun. Lett..

[48] Yang Y., Zhang B., Guo D., Xiong Z., Niyato D., Han Z. (2024). Can We Realize Data Freshness Optimization for Privacy Preserving-Mobile Crowdsensing with Artificial Noise?. IEEE Trans. Mob. Comput..

[49] Ju Y., Gao Z., Wang H., Liu L., Pei Q., Dong M., Mumtaz S., Leung V.C.M. (2024). Energy-Efficient Cooperative Secure Communications in mmWave Vehicular Networks Using Deep Recurrent Reinforcement Learning. IEEE Trans. Intell. Transp. Syst..

[50] Ju Y., Cao Z., Chen Y., Liu L., Pei Q., Mumtaz S., Dong M., Guizani M. (2024). NOMA-Assisted Secure Offloading for Vehicular Edge Computing Networks with Asynchronous Deep Reinforcement Learning. IEEE Trans. Intell. Transp. Syst..

[51] Crosara L., Laurenti N., Badia L. (2024). Age of information is not just a number: Status updates against an eavesdropping node. Ad Hoc Netw..

[52] Zhong J., Yates R.D. Timeliness in Lossless Block Coding. Proceedings of the Data Compression Conference (DCC).

[53] Zhong J., Yates R.D., Soljanin E. Timely Lossless Source Coding for Randomly Arriving Symbols. Proceedings of the IEEE Information Theory Workshop (ITW).

